# A selective hybrid fluorescent sensor for fructose detection based on a phenylboronic acid and BODIPY-based hydrophobicity probe†

**DOI:** 10.1039/d2ra01569b

**Published:** 2022-05-18

**Authors:** Gengo Kashiwazaki, Ryo Watanabe, Akihiro Nishikawa, Koyori Kawamura, Takashi Kitayama, Takao Hibi

**Affiliations:** Major in Advanced Bioscience, Graduate School of Agriculture, Kindai University 3327-204, Nakamachi Nara Nara 631-8505 Japan kitayama@nara.kindai.ac.jp; Department of Bioscience and Biotechnology, Faculty of Bioscience and Biotechnology, Fukui Prefectural University 4-1-1 Matsuoka-Kenjojima, Eiheiji Fukui 910-1195 Japan hibi@fpu.ac.jp

## Abstract

Fructose is widely used in the food industry. However, it may be involved in diseases by generating harmful advanced glycation end-products. We have designed and synthesized a novel fluorescent probe for fructose detection by combining a phenylboronic acid group with a BODIPY-based hydrophobicity probe. This probe showed a linear fluorescence response to d-fructose concentration in the range of 100–1000 μM, with a detection limit of 32 μM, which is advantageous for the simple and sensitive determination of fructose.

## Abstract 2

We have designed and synthesized a novel fluorescent probe for fructose detection through hydrophobic interactions by combining a phenylboronic acid group and a BODIPY-based hydrophobicity probe with a detection limit of 32 μM.

## Graphical abstract

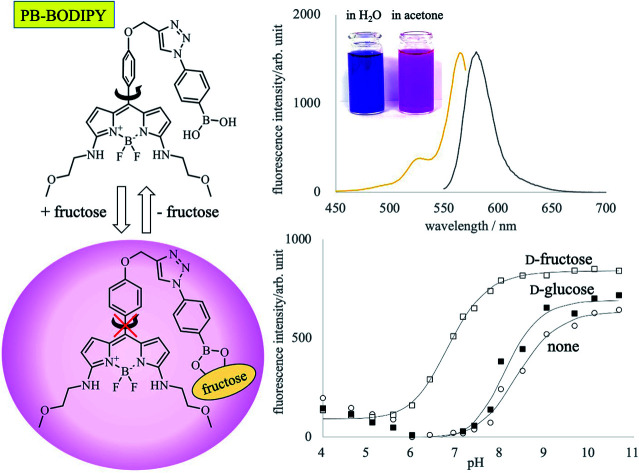

## Introduction

Fructose, a naturally occurring monosaccharide broadly found in fruits, has been increasingly used in the food industry because of its sweetness and its lack of inhibition of satiety compared with other sugars.^1^ At first, fructose was considered a safer sweetener than sucrose because it would not produce glucose in the blood and not be associated with obesity or non-alcoholic fatty liver disease (NAFLD), which is assumed to be caused primarily by high-fat diets. However, there have been several discussions on these speculations. In particular, fructose is a potent reducing sugar and may be involved in aging, atherosclerosis, and pathogenesis of the vascular, renal, and ocular complications of diabetes by producing toxic advanced glycation end-products.^2^ Furthermore, regarding obesity and NAFLD, the prevalence of these diseases did not improve even with a decrease in the percentage of calories ingested from saturated fatty acids, suggesting other dietary or environmental factors, and fructose can be one of the candidates of a leading factor of several metabolic disorders such as NAFLD^3,4^ and cardiometabolic disease.^5^ In addition, fructose has been suggested as a potential factor with adverse effects on blood pressure, hypertension, inflammation,^6^ and bowel disturbances.^2,7^ Therefore, a simple method for quickly detecting fructose would be beneficial and stimulate a wide range of research in this field. The quantitative detections of fructose have typically been achieved by the specificity of the enzymes^8^ or the chemical selectivity of phenylboronic acid (PB) as PB can covalently bond with *cis*-1,2- or 1,3-diol to form five- or six-membered cyclic boronic esters.^9–14^ The excellent molecular recognition of enzymes is manifested by the concerted action of multiple noncovalent interactions, such as hydrogen bonding, π–CH interactions, and hydrophobic interactions. Similarly, in chemical selection by PB, higher selectivity was expected to utilize hydrophobic interactions simultaneously. Here, we decided to construct a sensor molecule by combining a lipophilic fluorescence dye boron-dipyrromethene (BODIPY) with a phenylene linker because it would enhance the fluorescence as a result of inhibiting rotation of the phenyl group when bound to the analyte.^15^ This linker was connected to PB *via* triazole formed by click reaction, and two side chains further modified BODIPY to increase aqueous solubility.

BODIPY is suitable for conjugation with PB because of the relatively high photostability, high fluorescence quantum yield, and narrow absorption and emission spectra of the dye.^16–20^ The BODIPY skeleton also has an advantage in that it can be readily prepared by the well-established condensation methods of two pyrroles with various aryl aldehydes incorporated at the *meso*-position. In addition, PB-BODIPY probes can be used to detect sugars or polyols, including fructose,^21,22^ or bind to glycan chains of antibodies by a quartz crystal microbalance.^23^ The click reaction, a copper(i)-catalyzed azide–alkyne 1,3-dipolar cycloaddition, is a valuable conjugation method, and several azide- or ethynyl-substituted BODIPY derivatives were developed with azide at the α-position,^24,25^ directly^26^ at the *meso*-position, or propargyloxy group^27,28^ or tetrazine^29^ at the *meso*-phenylene.

Many environment-sensitive fluorescent probes have been developed and frequently used in biochemical research. For example, hydroxyazaphenanthrene was reported as a fluorescence probe for protein polarity^30^ and monodansylcadaverine as a solvent polarity probe.^31^ On the other hand, BODIPY has been considered a dye that is relatively insensitive to solvent polarity and pH change. Later in 2007, however, Sunahara and coworkers constructed a BODIPY library to investigate solvent polarity dependence and protein surface hydrophobicity and reported it as an environmental polarity sensor.^32^ Based on this idea of environmental sensitivity, Duan and coworkers synthesized two BODIPY dyes with an expanded solvent-polarity sensitive π-system and observed fluorescence enhancement with bovine serum albumin.^32^ Recently, Petrushenko and coworkers developed 8-CF_3_ BODIPY with four different substituents on the phenyl ring at the 3-position and discussed the on–off threshold in terms of intramolecular charge transfer.^33^

Interestingly, in our preliminary study on the HPsensor 2 for sensing protein surface-hydrophobicity,^15^ the regression analysis of the *E*^N^_T_ solvent polarity *vs. F*_max_ intensity plot provided two different linear regression lines (ESI Fig. S1†). One is from alcohols and the other from non-alcoholic solvents, indicating that this sensor can recognize the hydroxy group. In pursuit of a BODIPY derivative with higher recognition ability toward fructose among ketoses which are kinetically advantageous than aldoses, we designed and synthesized a clickable BODIPY scaffold with alkyne and conjugated with PB. The 2-methoxyethylamino group was introduced at the α-position of the two pyrroles to achieve higher aqueous solubility.^15^ With this fluorescent molecule, the sensitivity to solvent polarity was examined, pH condition was optimized, and the selectivity for saccharides was evaluated. Moreover, the titration curve of saccharides against the PB-BODIPY was determined.

## Materials and methods

### Materials

Fructose, mannose, and galactose were obtained from Nacalai Tesque, Inc. (Kyoto, Japan). Glucose, arabinose, sucrose, and norbixin standard were obtained from FUJIFILM Wako Pure Chemical Co., Ltd (Osaka, Japan). Allose and psicose were obtained from Tokyo Chemical Industry Co., Ltd (Tokyo, Japan). All other chemicals were of analytical-reagent grade and were used without further purification. All aqueous solutions were prepared using water obtained from a Milli-Q system (Millipore, Bedford, MA, USA).

Column chromatography was performed on silica gel (70–230 mesh). Thin-layer chromatography (TLC) was performed on Merck 60 F254 silica gel plates. NMR spectra were recorded on a Bruker instrument (AVANCE III Nanobay 400) at 400 MHz for ^1^H and 100 MHz for ^13^C or on a Bruker Nanobay 500 MHz spectrometer with tetramethylsilane (TMS) as the internal standard. Chemical shifts (*δ*) are reported in parts per million (ppm) from TMS. ^1^H NMR multiplicities are reported as: s = singlet, d = doublet, t = triplet, q = quartet, m = multiplet, and br = broad. Infrared spectra were recorded on a Shimadzu spectrophotometer (IRAffinity-1S). High-resolution mass spectrometry (HRMS) measurements were recorded on a Waters instrument (Q-TOF Premier). The chemicals were of commercially available reagent grade and used without purification. Pyrrole was distilled *in vacuo* before use.

### Synthesis of 4-propargyloxybenzaldehyde (2)^34^

Compound 2 was synthesized according to the reported procedure.^34^ Yield (75%, white solid). ^1^H NMR (CDCl_3_, 400 MHz): *δ* 9.91 (1H, s, CHO), 7.86 (2H, d, *J* = 8.8 Hz, CH at C3, C7), 7.10 (2H, d, *J* = 8.8 Hz, CH at C4, C6), 4.78 (2H, d, *J* = 2.4 Hz, CH_2_ at C8), 2.57 (1H, t, *J* = 2.4 Hz, CH at C10).

### Synthesis of 5-(4-propargyloxyphenyl)dipyrromethene (3)^35^

Compound 2 was synthesized according to the reported conditions.^36^ 0.18 M HCl aq. (50 mL), pyrrole (1.29 mL, 18.7 mmol), and 2 (1.00 g, 6.24 mmol) were added to a 300 mL three-necked eggplant flask, and the mixture was stirred at room temperature for 4 h. After adding saturated NaHCO_3_ aq., the solution was extracted with ethyl acetate (3 × 200 mL), the organic layer was washed with brine, and the organic layer was dried over anhydrous Na_2_SO_4_ followed by evaporation to dryness. The residue was purified by silica gel column chromatography (hexane/ethyl acetate = 8/1) to obtain 3 (1.01 g, 3.66 mmol, 59%) as a yellow oil. ^1^H NMR (CDCl_3_, 400 MHz): *δ* 7.92 (2H, br s, NH at N1, N2), 7.15 (2H, d, *J* = 8.4 Hz, CH at C12, C14), 6.93 (2H, d, *J* = 8.8 Hz, CH at C11, C15), 6.70 (2H, m, CH at C1, C9), 6.16 (2H, q, *J* = 2.9 Hz, CH at C2, C8), 5.91 (2H, m, CH at C3, C7), 5.44 (1H, s, CH at C5), 4.68 (2H, d, *J* = 2.4 Hz, CH_2_ at C16), 2.52 (1H, t, *J* = 2.4 Hz, CH at C18).

### Synthesis of 3,7-dichloro-5,5-difluoro-10-(4-(prop-2-yn-1-yloxy)phenyl)-5*H*-4λ^4^,5λ^4^-dipyrrolo[1,2-*c*:2′,1′-*f*][1,3,2]diazaborinine (4)

Compound 3 (270 mg, 0.98 mmol) in 5.0 mL dry THF was added to a 100 mL three-necked flask, purged with N_2_, and cooled to −78 °C. *N*-Chlorosuccinimide (287 mg, 2.15 mmol) in 25 mL THF was added dropwise to the cooled solution. The mixture was stirred at −78 °C for 15 min, and it was further stirred for 2 h at room temperature. Water was added, and the residue was extracted with CH_2_Cl_2_ (3 × 150 mL). The organic layer was dried over anhydrous Na_2_SO_4_, and the filtrate was evaporated to dryness. The subsequent oxidation was immediately conducted without purification. 2,3-Dichloro-5,6-dicyano-1,4-benzoquinone (266 mg, 1.17 mmol) was added to a solution of the reaction product in 30 mL CH_2_Cl_2_ in a 200 mL eggplant flask, and the mixture was stirred at room temperature for 2 h. *N*,*N*-Diisopropylethylamine (1.00 mL, 5.86 mmol) and boron trifluoride–diethyl ether complex 8.0 eq. (0.985 mL, 7.82 mmol) were added dropwise, and the mixture was stirred at room temperature for 24 h. After water was added to the mixture, the residue was extracted with CH_2_Cl_2_ (3 × 150 mL). The organic layer was dried over anhydrous Na_2_SO_4_, and the filtrate was evaporated to dryness. The residue was purified by flash column chromatography (hexane/ethyl acetate = 5/1) to obtain 4 (150 mg, 0.38 mmol, 39%) as a reddish brown solid. Mp: 201–204 °C. ^1^H NMR (CDCl_3_, 400 MHz): *δ* 7.46 (2H, d, *J* = 8.7 Hz, CH at C12, C14), 7.12 (2H, d, *J* = 8.7 Hz, CH at C11, C15), 6.89 (2H, d, *J* = 4.2 Hz, CH at C2, C8), 6.44 (2H, d, *J* = 4.2 Hz, CH at C3, C7), 4.79 (2H, d, *J* = 2.4 Hz, CH_2_ at C16), 2.60 (1H, t, *J* = 2.4 Hz, CH at C18). ^13^C{^1^H} NMR (CDCl_3_, 100 MHz): *δ* 159.9 (C13), 144.4 (C1, C9), 143.7 (C5), 133.7 (C4, C6), 132.2 (C12, C14), 131.5 (C2, C8), 125.6 (C10), 118.7 (C3, C7), 115.0 (C11, C15), 77.8 (C17), 76.3(C18), 56.0 (C16). These peaks were assigned by H–H correlation spectroscopy (COSY), hetero nuclear multiple quantum coherence (HMQC), and heteronuclear multiple bond coherence (HMBC). HRMS *m*/*z*: [M + Na]^+^ calcd for C_18_H_11_BF_2_N_2_OCl_2_ 413.0207; found 413.0187.

### Synthesis of 5,5-difluoro-*N*^3^,*N*^7^-bis(2-methoxyethyl)-10-(4-(prop-2-yn-1-yloxy)phenyl)-5*H*-4*λ*^4^,5*λ*^4^-dipyrrolo[1,2-*c*:2′,1′-*f*][1,3,2]diazaborinine-3,7-diamine (5)

Compound 4 (1.5 g, 3.84 mmol), 2-methoxyethylamine (2.67 mL, 30.7 mmol), and CH_3_CN (5.0 mL) were added to a 10.2 cm ace high pressure-resistant tube, and the mixture was stirred at 100 °C for 16 h. The solution was evaporated to dryness. The residue was purified by silica gel column chromatography (hexane/CH_2_Cl_2_/ethyl acetate = 3/2/1) to obtain 5 (1.04 g, 2.2 mmol, 58%) as a violet solid. Yield (58%: violet solid). Mp: 159–162 °C. IR (KBr): 3404, 3283, 2924, 1597, 1547, 1470, 1433, 1342, 1240 cm^−1^. ^1^H NMR (CDCl_3_, 400 MHz): *δ* 7.39 (2H, d, *J* = 8.7 Hz, CH at C12, C14), 7.02 (2H, d, *J* = 8.7 Hz, CH at C11, C15), 6.56 (2H, d, *J* = 4.2 Hz, CH at C2, C8), 5.74 (2H, d, *J* = 4.2 Hz, CH at C3, C7), 5.72 (2H, br s, NH at N3, N4), 4.74 (2H, d, *J* = 2.4 Hz, CH_2_ at C22), 3.59 (4H, t, *J* = 5.5 Hz, CH_2_ at C17, C20), 3.44 (4H, m, CH_2_ at C16, C19), 3.40 (6H, s, CH_3_ at C18, C21), 2.57 (1H, t, *J* = 2.4 Hz, CH at C24). ^13^C{^1^H} NMR (CDCl_3_, 100 MHz): *δ* 158.0 (C13), 156.5 (C1, C9), 131.6 (C12, C14), 131.5 (C5), 130.9 (C4, C6), 129.0 (C2, C8), 128.4 (C10), 114.3 (C11, C15), 101.0 (C3, C7), 78.4 (C23), 75.8 (C24), 71.1 (C17, C20), 59.0 (C18, C21), 55.9 (C22), 44.3 (C16, C19). These peaks were assigned by H–H COSY, HMQC, and HMBC. HRMS *m*/*z*: [M + Na]^+^ calcd for C_24_H_27_BF_2_N_4_O_3_ 491.2042; found 491.2036.

### Synthesis of (4-(4-((4-(5,5-difluoro-3,7-bis((2-methoxyethyl)amino)-5H-4λ^4^,5λ^4^-dipyrrolo[1,2-*c*:2′,1′-f][1,3,2]diazaborinin-10-yl)phenoxy)methyl)-1*H*-1,2,3-triazol-1-yl)phenyl)boronic acid (6)

CuI (4.0 mg, 21 μmol), DIPEA (7.4 μL, 43 μmol), acetic acid (2.4 μL, 43 μmol), 5 (100 mg, 214 μmol), 2-(4-azidophenyl)-5,5-dimethyl-1,3,2-dioxaborinane (59.0 mg, 256 μmol), and CH_2_Cl_2_ (0.5 mL) were added to a microtube, and the mixture was stirred at room temperature for 30 min. The solution was evaporated to dryness. The residue was purified by silica gel column chromatography (hexane/acetone/chloroform = 2/1/1) to obtain 6 (66.2 mg, 105 μmol, 48%) as a violet solid. Yield (48%: violet solid). Mp: 39–41 °C. IR (KBr): 3394, 1605, 1551, 1427, 1342, 1242, 1172, 1096 cm^−1^. ^1^H NMR (CDCl_3_, 500 MHz): *δ* 8.14 (1H, s, CH at C24), 7.94 (2H, d, *J* = 8.3 Hz, CH at C27, C29), 7.81 (2H, d, *J* = 8.3 Hz, CH at C26, C30), 7.40 (2H, d, *J* = 8.5 Hz, CH at C12, C14), 7.07 (2H, d, *J* = 8.7 Hz, CH at C11, C15), 6.56 (2H, d, *J* = 4.4 Hz, CH at C2, C8), 5.74 (2H, br s, NH at N3, N4), 5.74 (2H, br s, CH at C3, C7), 5.37 (2H, s, CH_2_ at C22), 4.86 (2H, s, OH at O4, O5), 3.59 (4H, t, *J* = 5.5 Hz, CH_2_ at C17, C20), 3.44 (4H, br s, CH_2_ at C16, C19), 3.40 (6H, s, CH_3_ at C18, C21). ^13^C{^1^H} NMR (CDCl_3_, 125 MHz): *δ* 158.6 (C13), 156.5 (C1, C9), 145.0 (C23), 138.9 (C28), 135.3 (C27, C29), 132.5 (C25), 131.7 (C12, C14), 129.0 (C5), 128.4 (C2, C8), 128.2 (C4, C6), 120.8 (C24), 119.8 (C26, C30), 114.3(C11, C15), 114.0(C10), 101.0 (C3, C7), 71.2 (C17, C20), 62.1 (C22), 59.0 (C18, C21), 44.2 (C16, C19). These peaks were assigned by H–H COSY, HMQC, and HMBC. HRMS *m*/*z*: [M + Na]^+^ calcd for C_30_H_33_B_2_F_2_N_7_O_5_, 654.2595; found, 654.2623.

### Apparatus

U-2900 UV-Vis spectrophotometer (Hitachi High-Tech Science, Japan) was used to measure absorption spectra. Fluorescence spectra were recorded with a fluorescence spectrometer F-7000 (Hitachi High-Tech Science, Japan).

### Spectroscopic studies

The absorption spectra of PB-BODIPY dye solution in acetone were measured from 340 nm to 640 nm in acetone at 1 nm intervals. The fluorescent dye solution was freshly prepared before use. Relative fluorescence quantum yield *Φ*_f_ of PB-BODIPY dye was obtained by means of the method using the Parker–Rees relationship.^37^ The fluorescence standard used was rhodamine B dye (*Φ*_f_ = 0.97 using an excitation wavelength of 543 nm in ethanol). The emission spectra for the fluorescent dye were measured at 1 nm intervals using excitation wavelengths of 564 nm with both excitation and emission band widths at 2.5 nm.

Unless otherwise described, all experiments were conducted at 25 °C.

### Determination of saccharide concentration

All saccharide samples were weighed and dissolved with water, shaken vigorously, and diluted with 0.1 mol L^−1^ sodium phosphate buffer, pH 7.5. For the saccharide detection, PB-BODIPY in acetone (50 μmol L^−1^) was diluted with 0.1 mol L^−1^ sodium phosphate buffer solution (pH 7.4) to obtain the final 0.5 μmol L^−1^ dye solutions. For the recovery measurements, a sample mixture was prepared by adding 500 μL of a known concentration of fructose standard solution to 500 μL of test solution and 4 mL of sodium phosphate buffer solution, and then added the dye solution. Each different concentration of saccharide solution in the phosphate buffer was mixed with the dye solution, respectively, and then the mixture was transferred into a quartz cuvette for fluorescence measurement using a fluorescence spectrometer F-7000 (*λ*_ex_ = 559 nm, *λ*_em_ = 580 nm; excitation slit width, 2.5 nm; emission slit width, 2.5 nm; scanning speed, 720 nm min^−1^). For the pH response, PB-BODIPY acetone solution was diluted by different pH values of buffer solutions (pH 4–6, 0.1 mol L^−1^ sodium acetate buffer; pH 6.5–7.8, 0.1 mol L^−1^ sodium phosphate buffer; pH 8–9, 0.1 mol L^−1^ sodium borate buffer; pH 9.5–10.5, 0.1 mol L^−1^ sodium carbonate buffer) to obtain 0.5 μmol L^−1^ PB-BODIPY solution. It is noted that, instead of the borate buffer, 0.1 mol L^−1^ Tris/HCl buffer was used for the saccharide detection in the pH range of 8–9 to prevent inhibiting the formation of borate ester. The enzyme assay of d-fructose was conducted using fructose colorimetric/fluorometric assay kit (BioVision Inc., Mountain View, CA, USA) according to the manufacturer's instruction.

## Results

### Synthesis and characterization of PB-BODIPY

**Fig. 1:**
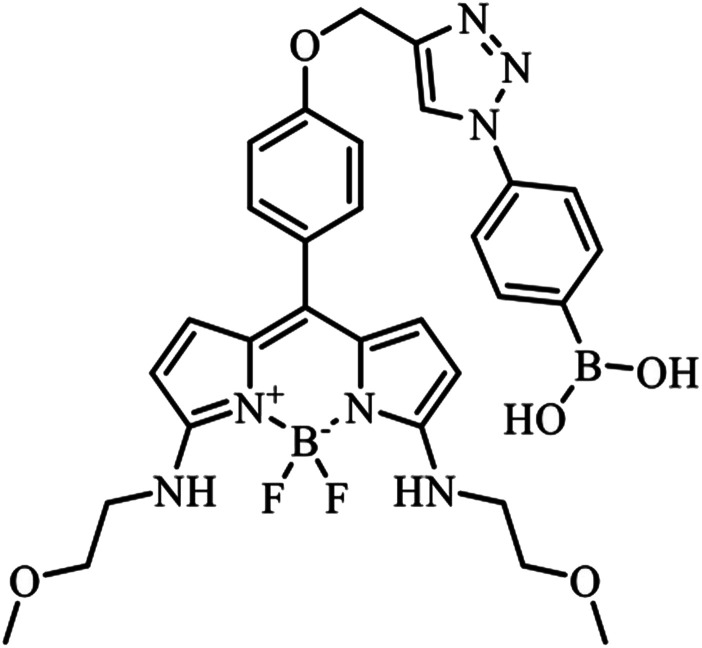
PB-BODIPY.

PB-BODIPY (Fig. 1) was synthesized according to the procedure described in Materials and methods. The molar absorption coefficient and the quantum yield of the purified dye were determined to be 1.05 × 10^5^ M^−1^ cm^−1^ (*λ*_abs_ = 565 nm) and 0.39 in acetone.

**Fig. 2:**
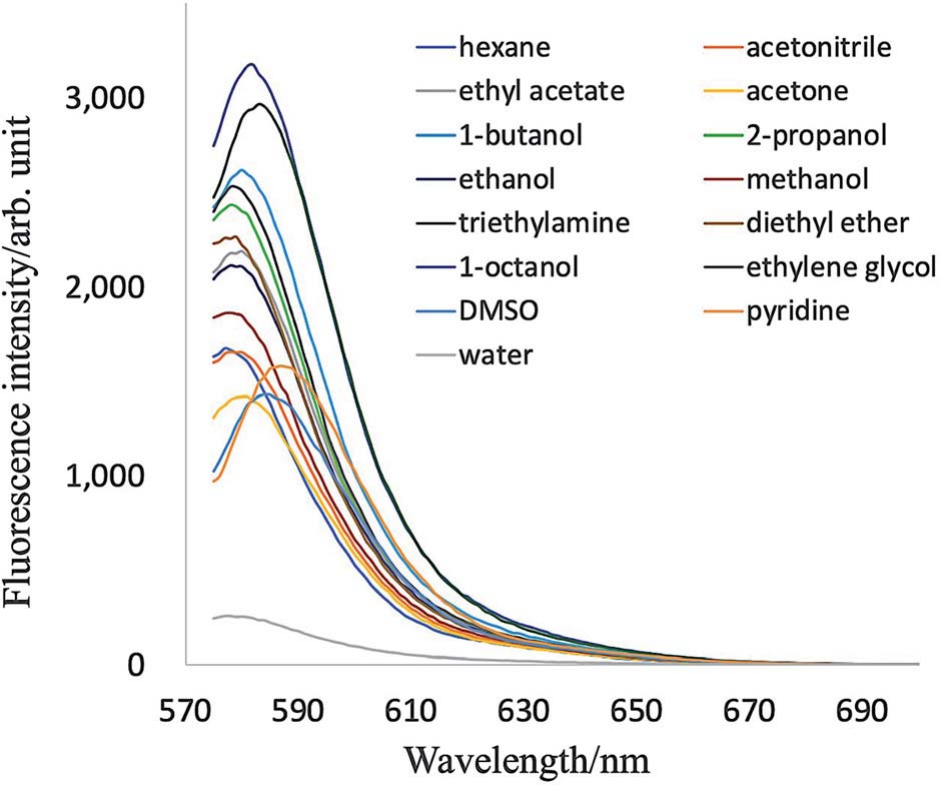
Fluorescence emission spectrum of PB-BODIPY in various solvents.

Fluorescence emission spectra of PB-BODIPY in some selected solvents are shown in Fig. 2. The dye exhibited only a small (10 nm or less) emission peak shift in the selected solvents with various polarities. The changes of related Stokes shifts were less than 5 nm in going from apolar to polar solvents.

**Fig. 3:**
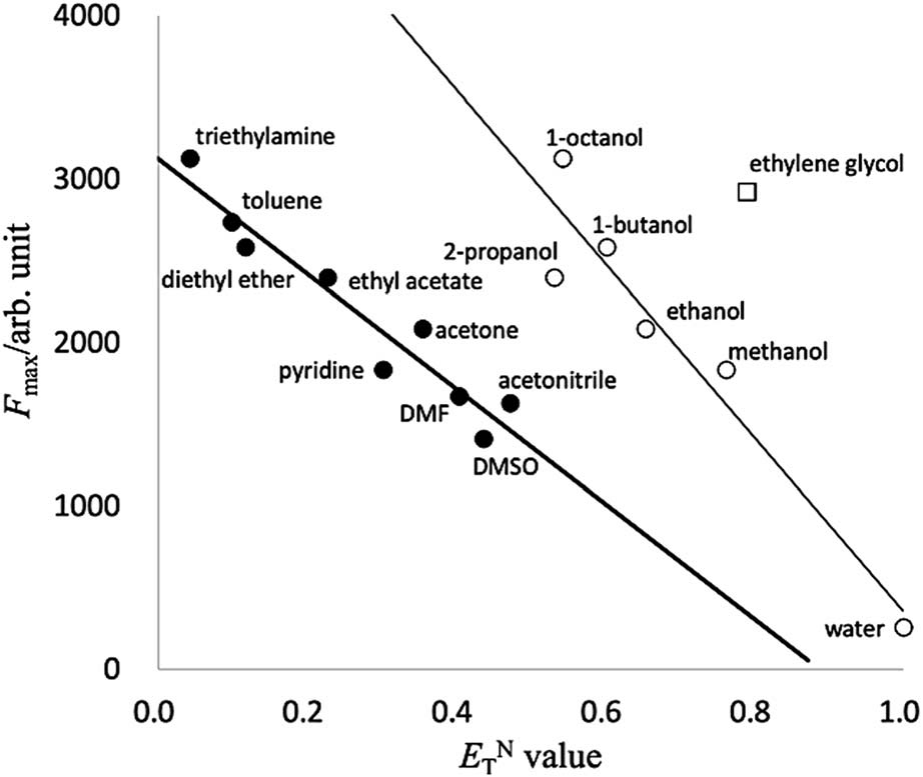
A correlation plot of the fluorescence intensities of PB-BODIPY *vs.* the *E*^N^_T_ value of the respective solvent.

To estimate the relation between solvent polarity and spectral properties, we compared the fluorescence intensities to the standard *E*^N^_T_ polarity scale of the selected solvents.^38^ A correlation plot of the fluorescence intensities of PB-BODIPY *vs.* the *E*^N^_T_ value of the respective solvent is shown in Fig. 3. Linear regression was used to fit the correlation relationship between the fluorescence intensities and *E*^N^_T_ value, where the fitted relationships for mono-alcohols (thin line) and other solvents (thick line) are *y* = 5675 − 5323*x*, *r*^2^ = 0.92 and *y* = 3123 − 3522*x*, *r*^2^ = 0.92, respectively. Fig. 3 suggested that the fluorescent emission intensity of ethylene glycol could be higher than mono-alcohol or other solvents with the same *E*^N^_T_ value. It is noted that HPsensor 2 dye, which has no boronic acid group, also showed a similar tendency of the correlation relationship (ESI Fig. S1†).

### pH dependency of fluorescent intensity

In an aqueous solution, phenylboronic acid reacts with water to form the boronate anion, and the p*K*_a_ value of phenylboronic acid is about 8.8.^39,40^ Boronic acid groups form fast and reversible covalent interactions with saccharides.^41^ When the boronic acids are complexed with a saccharide, its p*K*_a_ decreases and induces the anionic form of the boronic acid group at specific pH. When the boronic acids are conjugated with a fluorophore, the neutral form of the boronic acid group acts as an electron-withdrawing group while the anionic form acts as an electron-donating group. This change in the electronic properties of the boronic acid group can lead to spectral changes of the fluorophore.

**Fig. 4:**
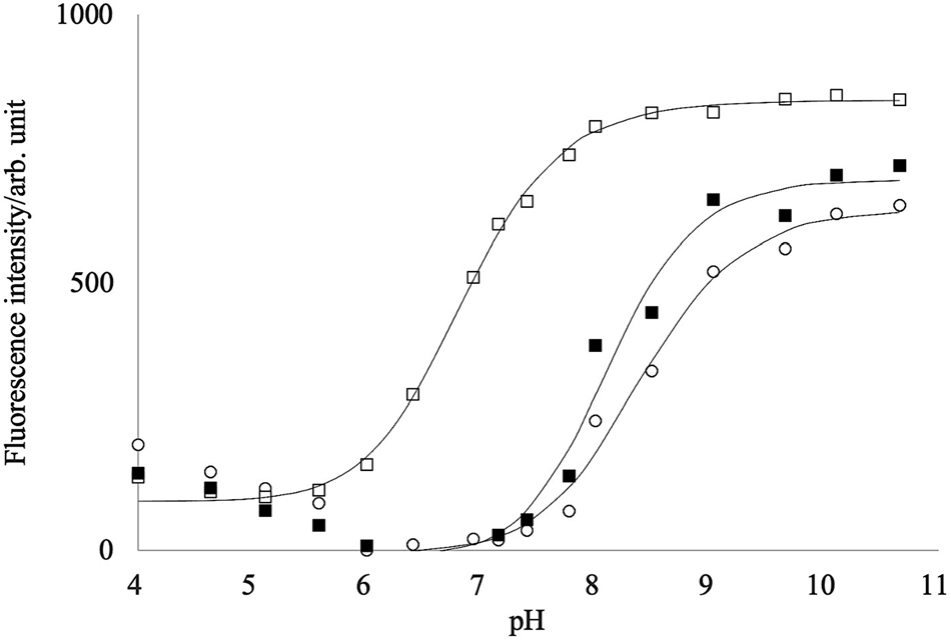
pH dependency of the fluorescence intensities of PB-BODIPY in the presence of d-glucose (■)/d-fructose (□), or without sugars (○).

Fig. 4 shows the fluorescence intensity change of PB-BODIPY with the pH change. A significant increase in the intensity was observed as the pH increases, although the emission peak shifts were negligible (data not shown). A theoretical fit to the titration curve yielded a p*K*_a_ of 8.4.

It has been reported that p*K*_a_ of the boronic acid–saccharide complex was lower than the free boronic acid.^39,42^ The fittings of the titration curves in Fig. 4 provided p*K*_a_s of 6.8 and 8.1 in the presence of 0.2 mol L^−1^ fructose and glucose, respectively. At pH 7.2–7.8, maximum changes of the titration curves with and without fructose were obtained, implying that the neutral form of PB-BODIPY is predominant in the absence of fructose and the anionic form of the dye–fructose complex in the presence of fructose. On the other hand, the addition of glucose did not change the fluorescence intensities much in this pH range, suggesting that the complex with glucose was not formed. Unless otherwise described below, our measurements at pH 7.4 were performed for all saccharides.

### Selectivity for saccharides

**Fig. 5:**
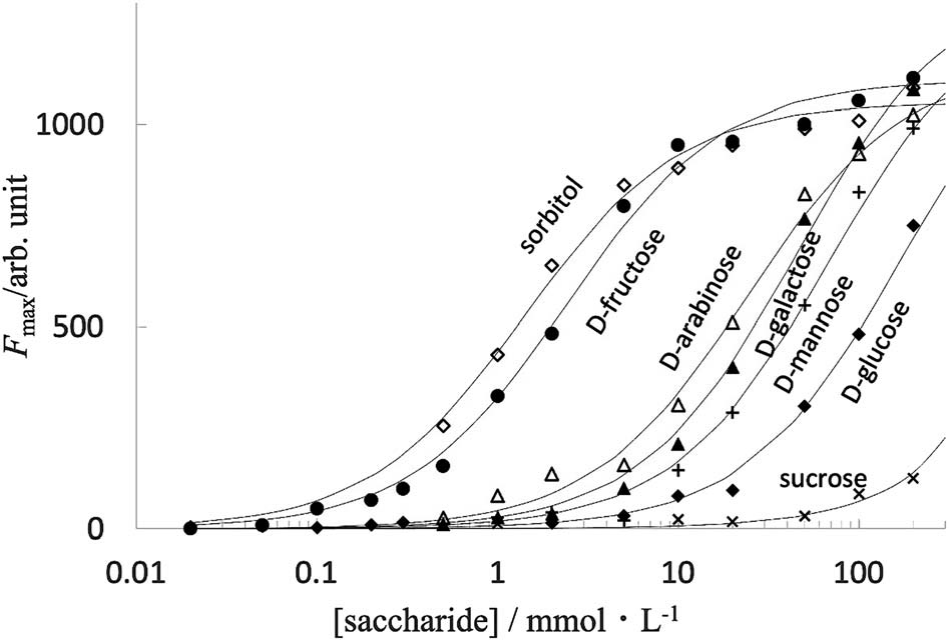
Selectivity for saccharides.

**Scheme 1:**
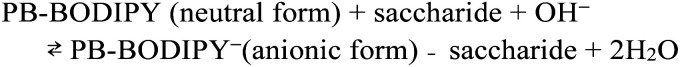


Fig. 5 shows the fluorescence intensity of PB-BODIPY at pH 7.4 at 25 °C as a function of the concentration of the saccharides (titration curves). Assuming that the boronic acid–diol complex formation follows Scheme 1, the association constant of the boronic acid–diol complex (*K*) can be estimated by titration of the saccharide with the dye.

In this case, the titration curve follows eqn (1).1
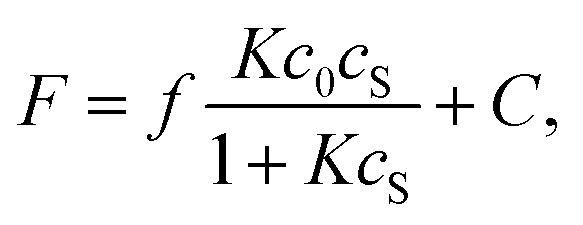
where *c*_0_ and *c*_S_ (*c*_0_ ≪ *c*_S_) are the initial concentration of BA-BODPY and saccharide, *f* is a proportionality factor of the fluorescence intensity, and *C* is a fluorescent intensity without saccharide.

The extent to which the diol moiety changes the fluorescence intensity depends on the binding affinity between boronic acid and diol. The association constants are: d-fructose (log *K* = 2.6); sorbitol (log *K* = 2.9); d-glucose (log *K* = 0.75); d-arabinose (log *K* = 1.6); d-mannose (log *K* = 1.2) and d-galactose (log *K* = 1.3) (sucrose is below the detection limit). Although the relative stability order indicates the inherent selectivity of all monoboronic acids,^43^ the log *K* difference between d-fructose and d-glucose is about 1.9, which to our best knowledge is one of the highest values among the known monoboronic acid-derivative. These results suggested that the titration of d-fructose using PB-BODIPY was not affected in the presence of 1 mmol L^−1^ aldose.

At sugar concentrations where *Kc*_S_ in eqn (1) is sufficiently smaller than 1, the equation can be approximated as follows.

1′ *F* = *fKc*_0_*c*_S_ + *C*.

**Fig. 6:**
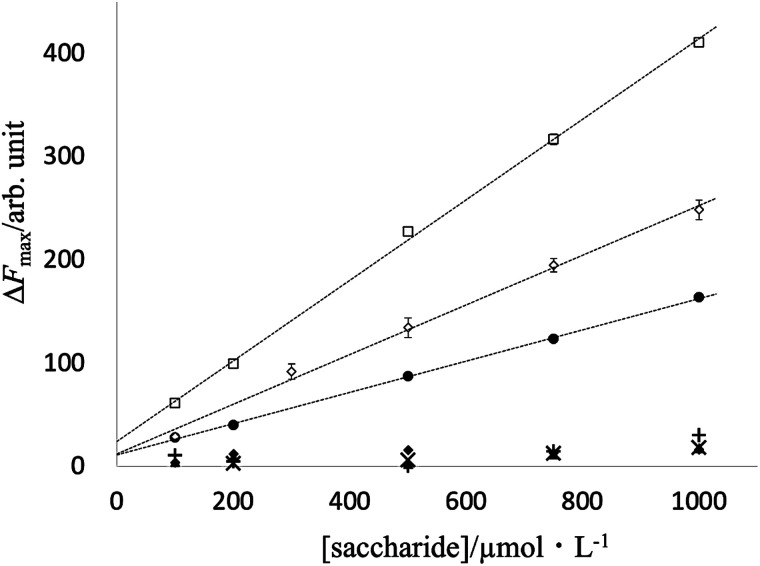
Fluorescence response at different concentrations of saccharides: (●) d-fructose; (□) d-psicose; (◇) d-sorbitol; (◆) d-glucose; (▲) d-galactose; (+) d-mannose; (×) d-allose.

In fact, for d-fructose and d-psicose as ketoses and the sugar alcohol d-sorbitol, the fluorescence intensity was approximately proportional to the sugar concentration in the range of 100 μmol L^−1^ to 1 mmol L^−1^; for the four aldoses, the fluorescence intensity was not sufficient to confirm linearity in the concentration range studied (Fig. 6).

Fluorescence linearity and detection limits for d-fructose, d-psicose, and sorbitol were analyzed for a PB-BODIPY of 0.5 μmol L^−1^ at 25 °C. d-Fructose was determined from 100 to 1000 μmol L^−1^ (*n* = 5) with a linear least-squares slope of 0.152 ± 0.002, an intercept of 11.0 ± 1.3, a correlation coefficient of 0.9997, and the detection limit of 32 μmol L^−1^.

d-Psicose was determined from 100 to 1000 μmol L^−1^ (*n* = 5) with a linear least-squares slope of 0.390 ± 0.008, an intercept of 24.0 ± 4.6, a correlation coefficient of 0.9995, and the detection limit of 43 μmol L^−1^. d-Sorbitol was determined from 100 to 1000 μmol L^−1^ (*n* = 5) with a linear least-squares slope of 0.241 ± 0.010, d-sorbitol was determined from 100 to 1000 μmol L^−1^ (*n* = 5) with a linear least-squares slope of 0.241 ± 0.010, an intercept of 12.0 ± 5.9, a correlation coefficient of 0.9976, and the detection limit of 85 μmol L^−1^.

The recovery measurements were performed to assess the overall accuracy of the present method. The based samples of known concentrations were spiked with a series of standard fructose solutions to the final concentrations covered the low, medium, and high levels. The results shown in Table 1 reveal that the recovery rates are 96.9–99.9%. The concentrations of 300, 700, and 1000 μmol L^−1^ of each d-fructose solution were analyzed to evaluate the intra- and inter-day reliability of the method. Intra-day precision was assessed by calculating the standard deviation (SD) and the coefficient of variation (CV%) values obtained by repeating the assay five times on the same day, while inter-day precision was carried out on four different days. The results are shown in Table 2. The intra-day and inter-day CV% values were less than 3 and 7 for all concentrations, suggesting satisfactory intra-day repeatability and recovery for fructose determination by the presented method.

**Table 1:** Spike recovery study conducted to validate the fluorescence assay using PB-BODIPY

Added fructose concentration (mmol L^−1^)	Measured fructose conc. (mmol L^−1^)	Recovery (%)
2	1.998	99.9
3	2.983	99.4
5	4.843	96.9

**Table 2:** Intra- and inter-day assay coefficients of variation (CVs) and total CV of the 3 different concentrations of fructose samples determined with the fluorescence assay using PB-BODIPY

Final fructose concentration (μmol L^−1^)	Intra-day			Inter-day^b^			CV_total_^c^ (%)
	df^a^	SD^a^ (μmol L^−1^)	CV (%)	df^a^	SD^a^ (μmol L^−1^)	CV (%)	
300	19	9.4	2.34	4	28.8	7.21	6.20
700	20	18.5	2.64	4	19.2	2.74	3.60
1000	18	21.5	2.15	4	62.5	6.25	4.79

a df = degree of freedom for each sample, SD = standard deviation.

b For inter-day assay CV, 3 fructose samples were analyzed in duplicate in 5 different runs carried out on 5 different days.

c CV_total_ was calculated by the following equation; 
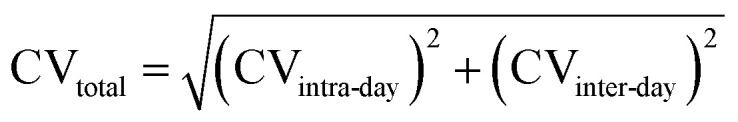
.

Determination of d-fructose in commercial foods and biological samples generally involves interference by several substances, such as reducing agents, alcohols, and proteins. The recoveries of 2 mmol L^−1^d-fructose were investigated in the presence of interfering substances (Table 3). As a result, 0.2 mol L^−1^ NaHCO_3_, 0.2 mol L^−1^ CH_3_COONa, and 0.2 mmol L^−1^ uric acid did not show any significant interferences. In the presence of 2% (v/v) ethanol and 2 μg mL^−1^ norbixin, increases of 19.1% and 8.9% were observed, respectively. These increases were probably attributed to the hydrophobicity of the interfering substance. Ascorbic acid showed a higher positive interference of more than 100%, probably because it is a six-carbon sugar acid with a polyol structure. Ovalbumin showed significant positive interferences, suggesting that the deproteinization pretreatment is necessary to apply PB-BODIPY to the fructose assay (ESI Table S1†).

**Table 3:** Fructose concentrations and recoveries measured in the presence of interfering substances

Interfering substance	Added fructose conc. (mmol L^−1^)	Measured fructose conc. (mmol L^−1^)	Recovery (%)
0.2 mol L^−1^ NaHCO_3_	2	2.178	108.9
0.2 mol L^−1^ CH_3_COONa	2	1.997	99.9
0.2 mmol L^−1^ uric acid	2	1.835	91.8
2 mmol L^−1^ ascorbic acid	2	4.584	229.2
2% (v/v) ethanol	2	2.381	119.1
2 μg mL^−1^ norbixin	2	2.178	108.9

## Discussion

In order to design a boronic acid-based fluorescence probe for saccharide sensing, the photochemical properties of the dye should be switched by the interaction with the analyte. Internal charge transfer (ICT)^22,44,45^ and photo-induced electron transfer (PET)^46,47^ mechanisms have been used as a switch, and many sensitive methods for the fluorescence detection of saccharides have been reported.^48,49^

Since the *cis*-1,2- and -1,3-diols are mostly ubiquitous to saccharides, the boronic acid can form stable cyclic boronic esters with various saccharides.^41^ Although d-fructose forms more stable complexes than d-glucose and other aldoses, the fructose sensor with a boronic acid did not have enough selectivity and sensitivity. In general, saccharides have been suggested to show hydrophobic properties, though they are highly water-soluble and insoluble in organic solvents. C. Buttersack estimated the hydrophobic interaction of carbohydrates using a C_18_-modified silica gel column and showed the hydrophobic row of the aldoses and ketoses, suggesting that d-fructose was more hydrophobic than d-glucose and the other aldohexoses.^50^ This gave us the idea to make a new fructose sensor by combining a hydrophobicity probe with phenylboronic acid.

HPsensor 2, developed by N. Dorh *et al.*, is an excellent hydrophobic probe with a 10- to 60-fold higher signal intensity than ANS.^15^ The fluorescence increase in the low polarity environment has been explained by the inhibition of free rotation of the aryl substituents leading to a decrease in non-radioactive decay. Interestingly, we unexpectedly found that the fluorescence of HPsensor 2 tended to be enhanced explicitly in the presence of alcohol (ESI Fig. S1†).

To emit strong fluorescence by introducing a phenylboronic acid group into HPsensor 2, PB-BODIPY was designed and synthesized as a fluorescent probe interacting with fructose. The obtained PB-BODIPY specifically interacts with fructose against aldoses at neutral pH and shows excellent properties as a selective fluorescent probe for fructose (Fig. 6). The fluorescence intensity for 1 mmol L^−1^d-fructose was 43 and 11 times higher than that of d-glucose or d-galactose at the same concentration, showing superior selectivity than the previously reported fluorescent probes for fructose.

Mixing fructose with PB-BODIPY resulted in a linear fluorescence response to the low d-fructose concentration; d-fructose concentration was measured from 100 to 1000 μmol L^−1^, and the detection limit was 32 μmol L^−1^. Therefore, PB-BODIPY is an advantageous fluorescent probe for sensitive and straightforward determination of the fructose concentration. Though the detection mechanism remains unclear, this fluorescent response should be caused at least partly by inhibiting the rotation of the phenyl group at the *meso*-position of BODIPY, which is one of the characteristics of the PB-BODIPY, and ICT or PET mechanism might also be involved. Since the interference by proteins and lipids has been a problem yet, some sample pretreatments will be required for the food and clinical analysis application, but this is currently under investigation and will be reported before long. In addition, this probe has the feature of replacing the recognition group of sugar with a click reaction. This probe is expected to be applied to determine oligosaccharides with higher hydrophobicity in the near future.

## Conclusions

The described PB-BODIPY probe can offer a rapid method for the specific determination of fructose. One of the probe characters introduces various functional groups other than boronic acid by using the click reaction. Based on the results obtained so far, this probe developed for fructose shows significant potential for determining oligosaccharides. Research in this direction is in progress.

## Author contributions

G. K. conducted conceptualization, wrote the original draft, and reviewed the whole draft. R. W. established the syntheses of the compounds, and reviewed the draft. A. N. synthesized the compounds and wrote the original draft. K. K. conducted formal analysis and validation. T. K. conducted project administration, review, and editing of the draft. T. H. conducted conceptualization, formal analysis, funding acquisition, investigation, project administration, review, and editing of the draft.

## Conflicts of interest

There are no conflicts to declare.

## Supplementary Material

RA-012-D2RA01569B-s001
