# Supplementary material for: A selective hybrid fluorescent sensor for fructose detection based on a phenylboronic acid and BODIPY-based hydrophobicity probe
Source: RSC Adv. 2022 May 18;12(24):15083–90. doi: 10.1039/d2ra01569b (PMC9116957; doi:10.1039/d2ra01569b)
Supplement: RA-012-D2RA01569B-s001 [file RA-012-D2RA01569B-s001.pdf]

Electronic Supplementary Material (ESI) for RSC Advances.
This journal is © The Royal Society of Chemistry 2022

# Supporting Information

# A selective hybrid fluorescent sensor for fructose detection based on a pheylboronic acid and BODIPY-based hydrophobicity probe

Gengo Kashiwazaki,[a] Ryo Watanabe,[a] Akihiro Nishikawa,[a] Koyori Kawamura,[b] Takashi Kitayama,[a]* Takao Hibi[b]*

[a]Major in Advanced Bioscience, Graduate School of Agriculture, Kindai University, 3327-204, Nakamachi, Nara, Nara 631-8505, Japan
[b]4-1-1 Matsuoka-Kenjojima, Eiheiji, Fukui 910-1195, Japan.
To whom correspondence should be addressed. E-mail: kitayama@nara.kindai.ac.jp, and hibi@fpu.ac.jp

## Table of contents

A correlation plot of the fluorescence intensities of HPSensor2 vs. the $E_T^N$ value ……Figure S1
$^1$H NMR spectrum of compound **2** ……Figure S2
$^1$H NMR spectrum of compound **3** ……Figure S3
$^1$H NMR spectrum of compound **4** ……Figure S4
$^{13}$C{$^1$H} NMR spectrum of compound **4** ……Figure S5
$^1$H NMR spectrum of compound **5** ……Figure S6
$^{13}$C{$^1$H} NMR spectrum of compound **5** ……Figure S7
$^1$H NMR spectrum of compound **6** ……Figure S8
$^{13}$C{$^1$H} NMR spectrum of compound **6** ……Figure S9
Effect of interference substances on fructose assay ……Table S1

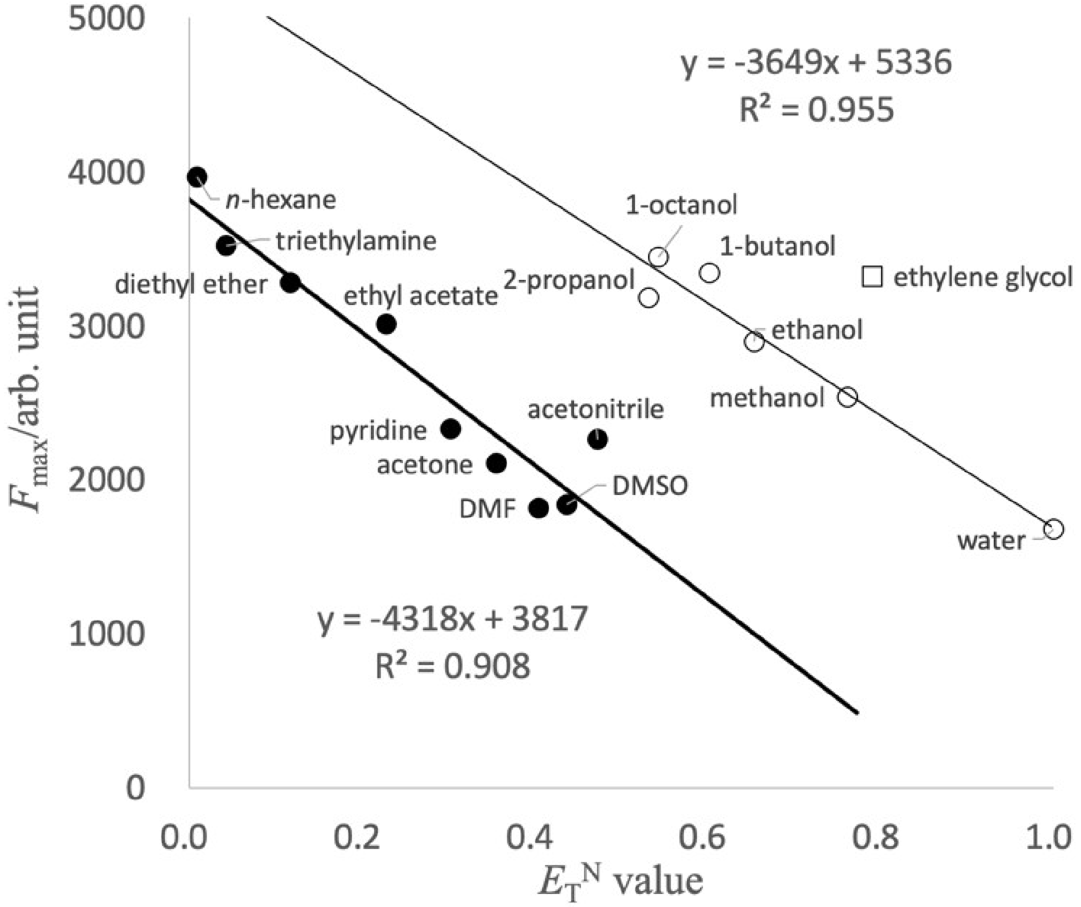


**Figure S1.** A correlation plot of the fluorescence intensities of HPSensor2 vs. the $E_T^N$ value of the respective solvent.

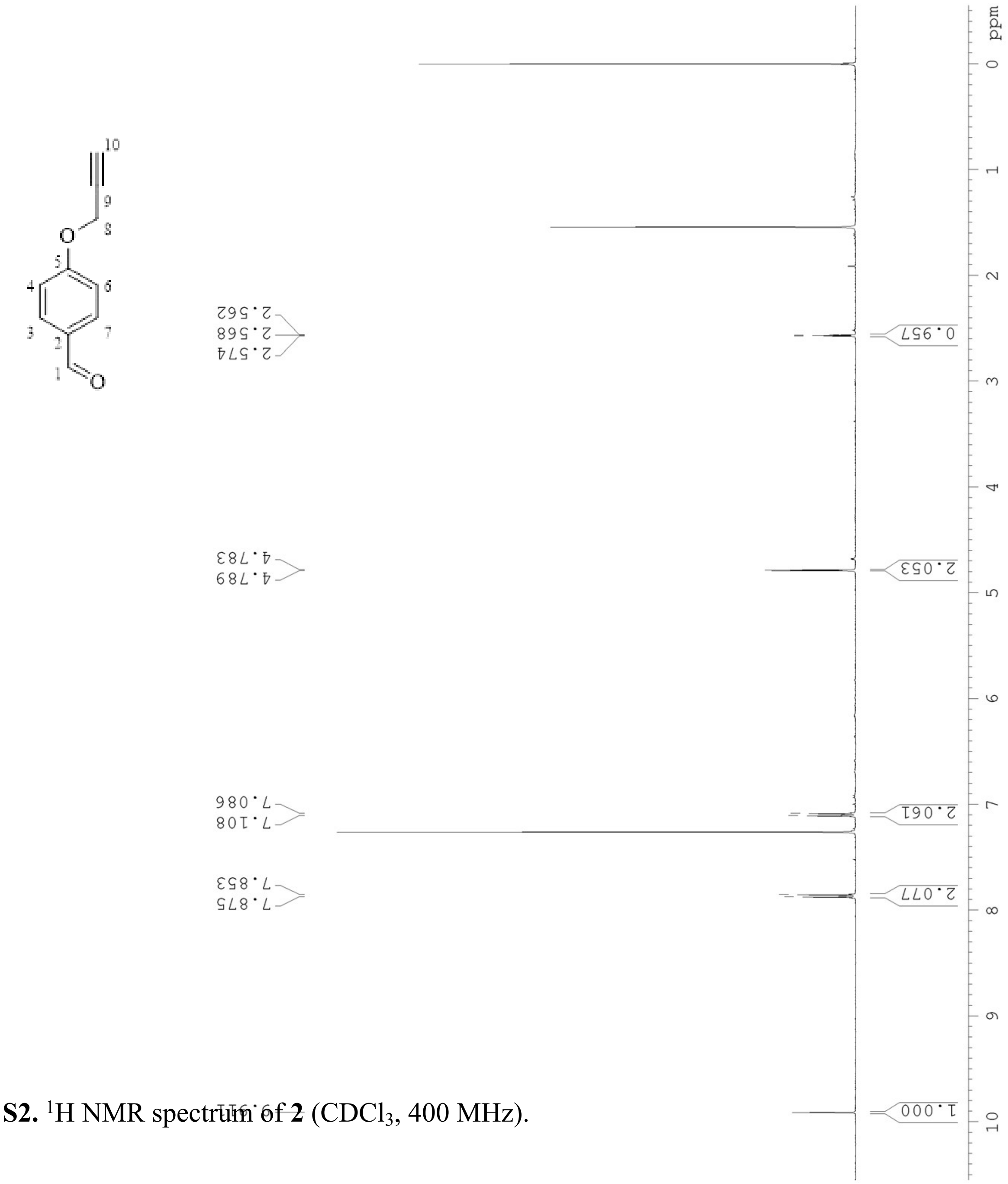


**Figure S2.** $^{1}$H NMR spectrum of **2** ($CDCl_3$, 400 MHz).

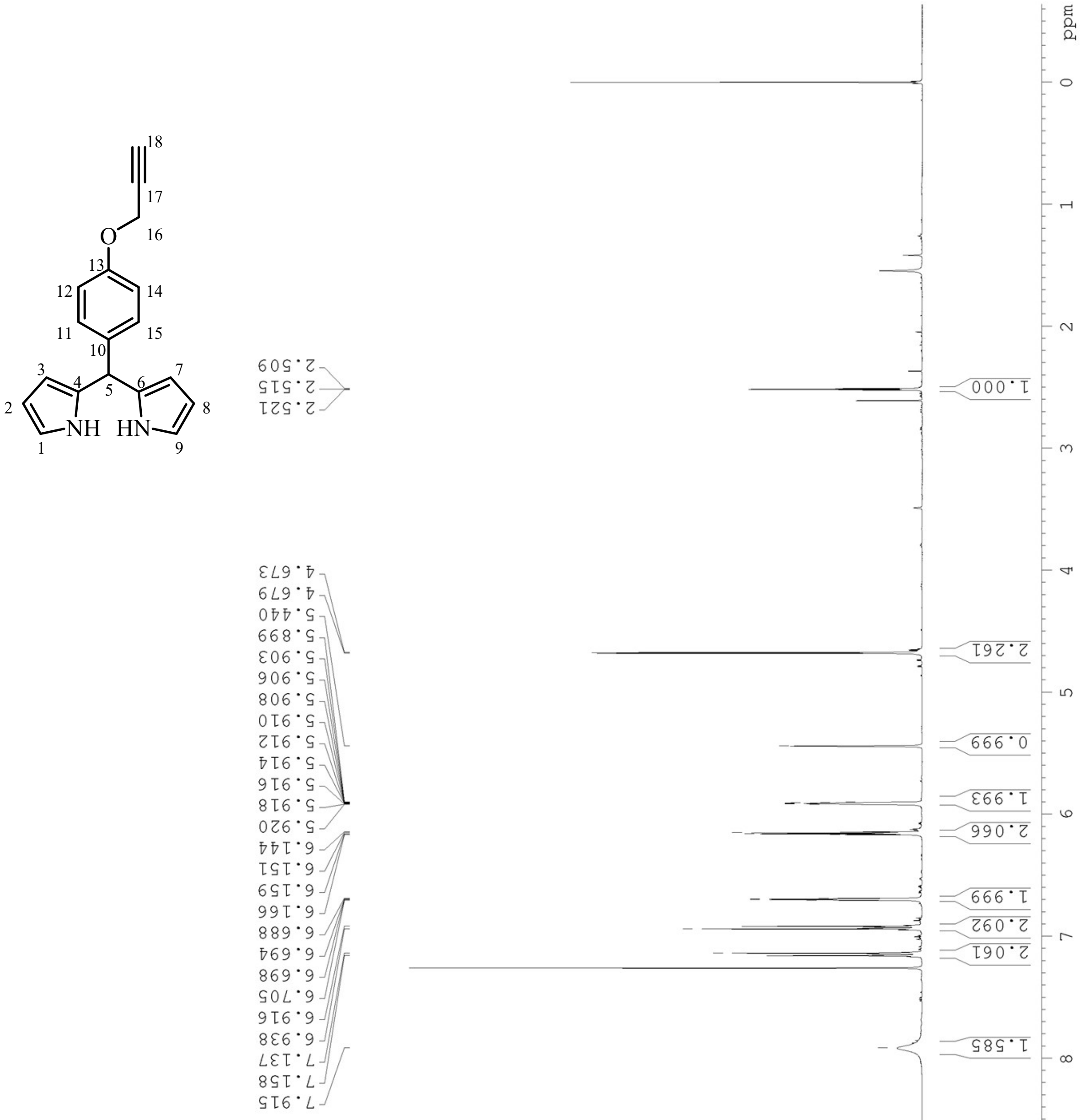


**Figure S3.** $^{1}$H NMR spectrum of **3** ($CDCl_3$, 400 MHz).

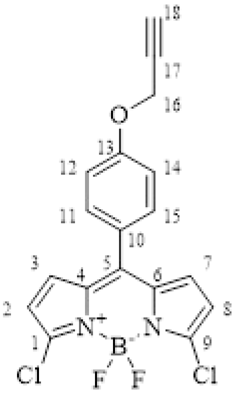


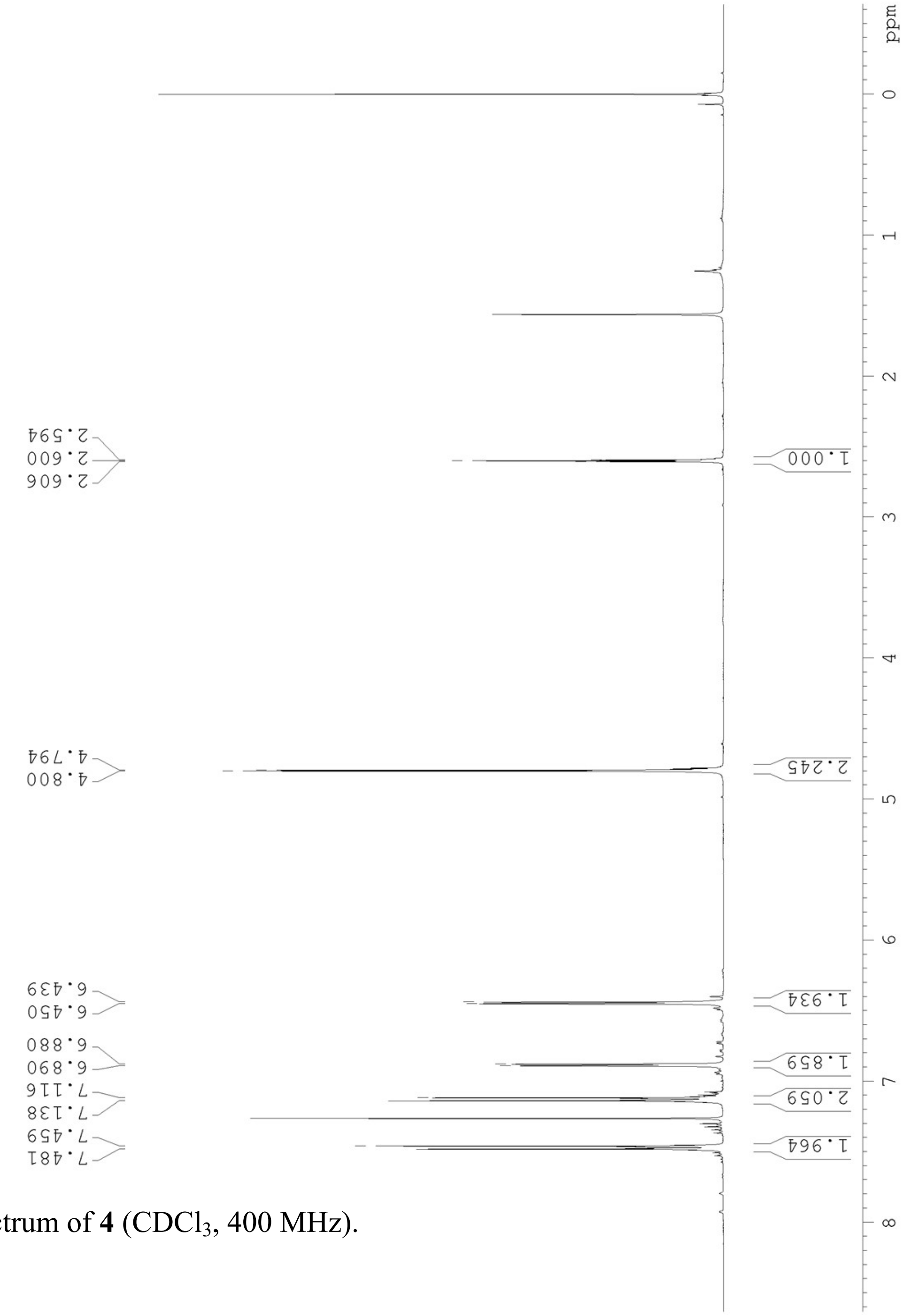


**Figure S4.** $^1$H NMR spectrum of **4** ($CDCl_3$, 400 MHz).

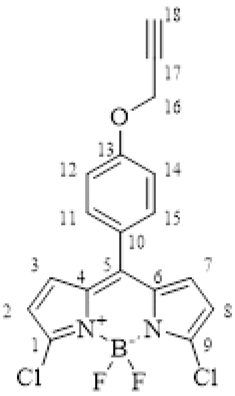

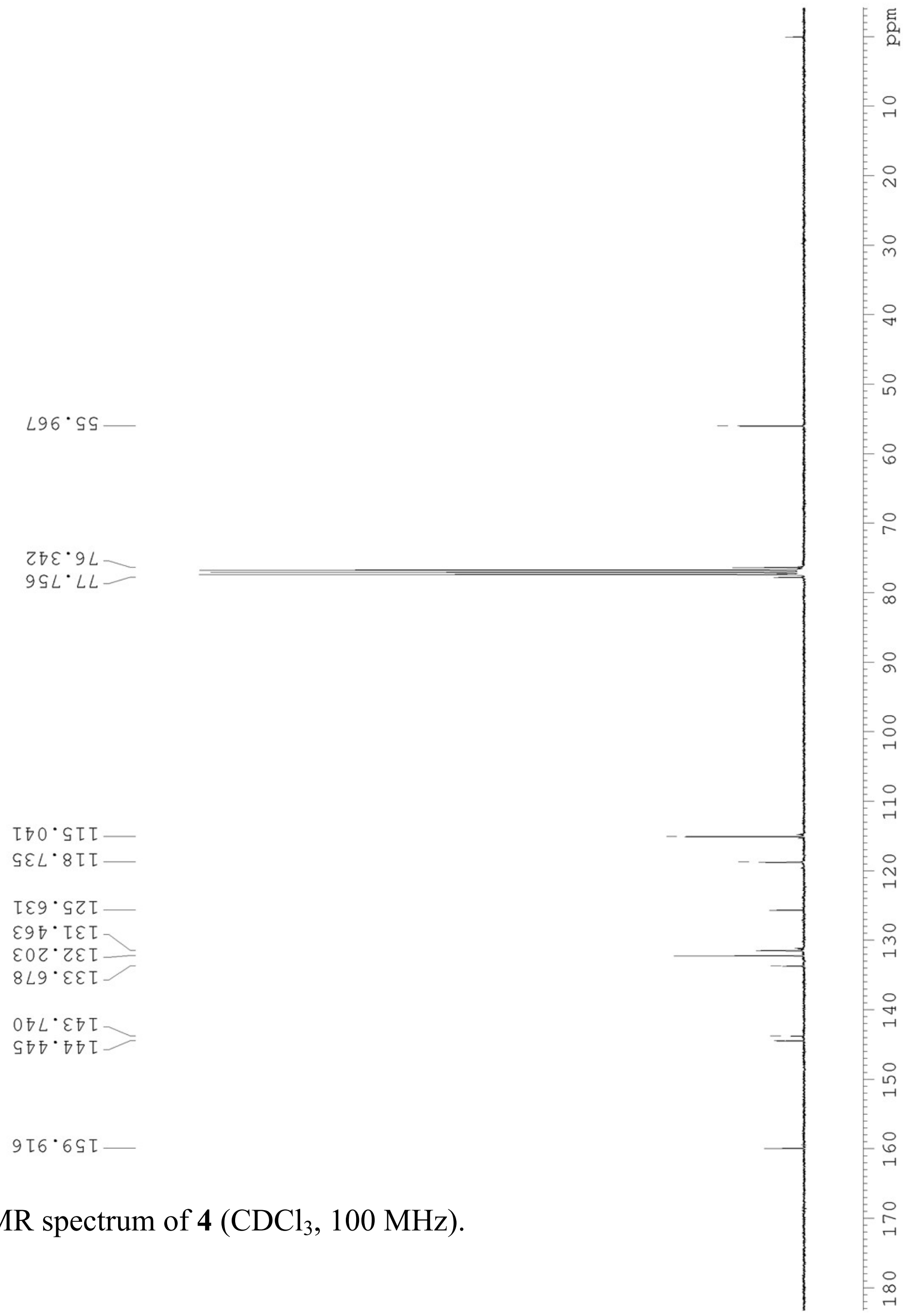


**Figure S5.** $^{13}C\{^1H\}$ NMR spectrum of **4** ($CDCl_3$, 100 MHz).

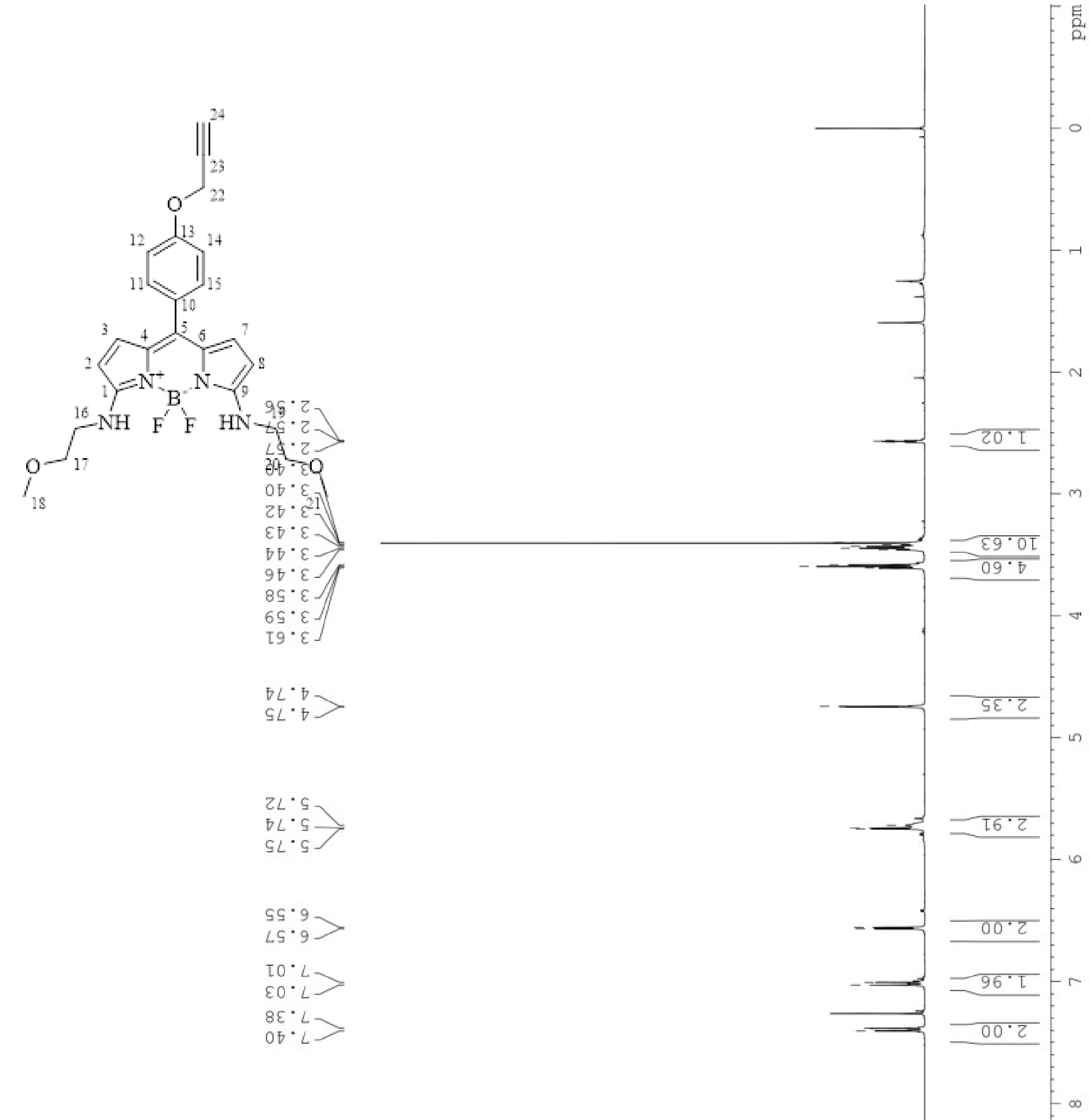


**Figure S6.** $^{1}H$ NMR spectrum of **5** ($CDCl_3$, 400 MHz).

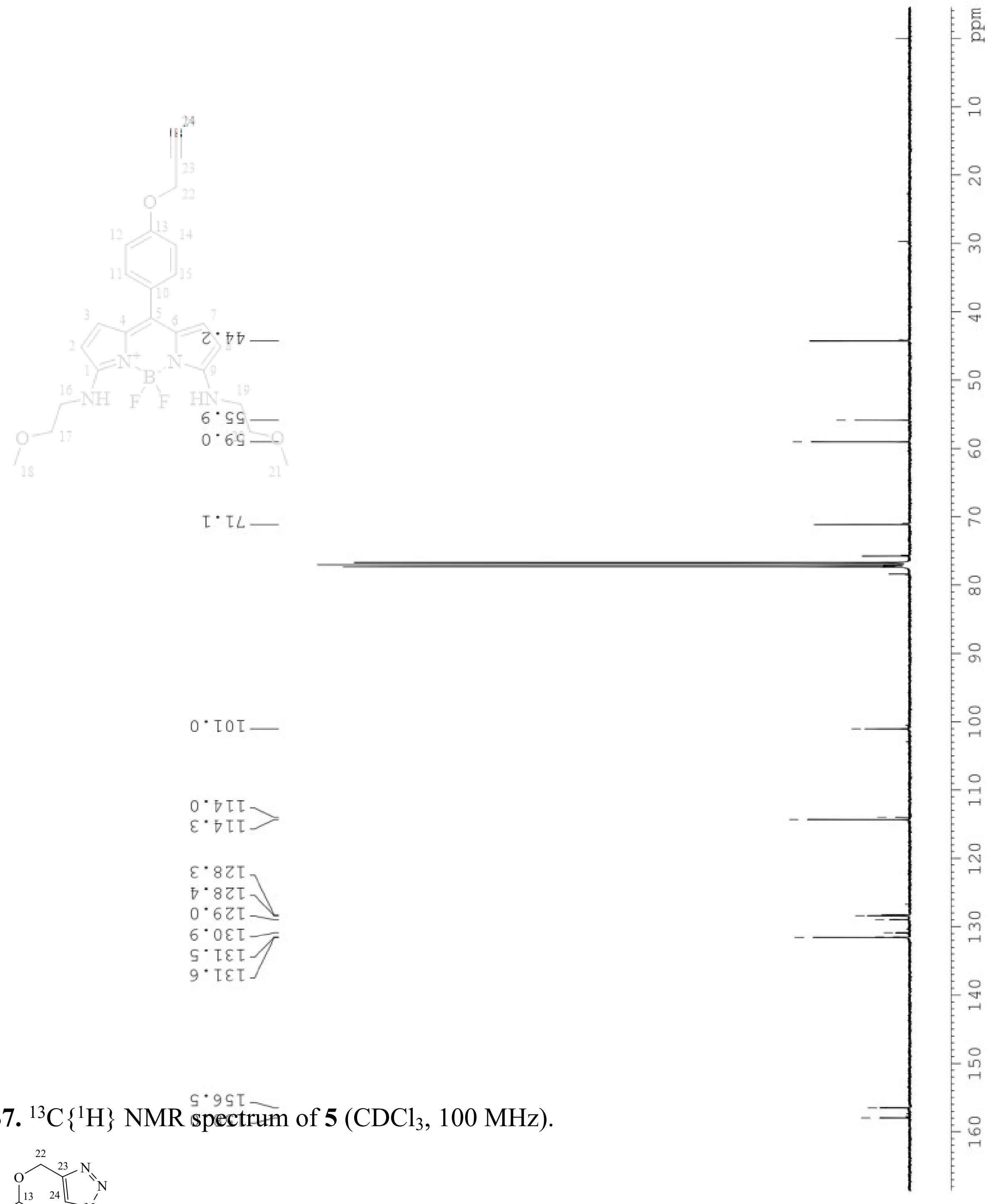


**Figure S7.** $^{13}C\{^{1}H\}$ NMR spectrum of **5** ($CDCl_3$, 100 MHz).

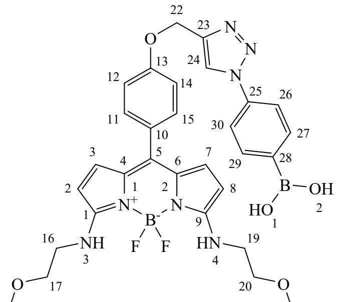

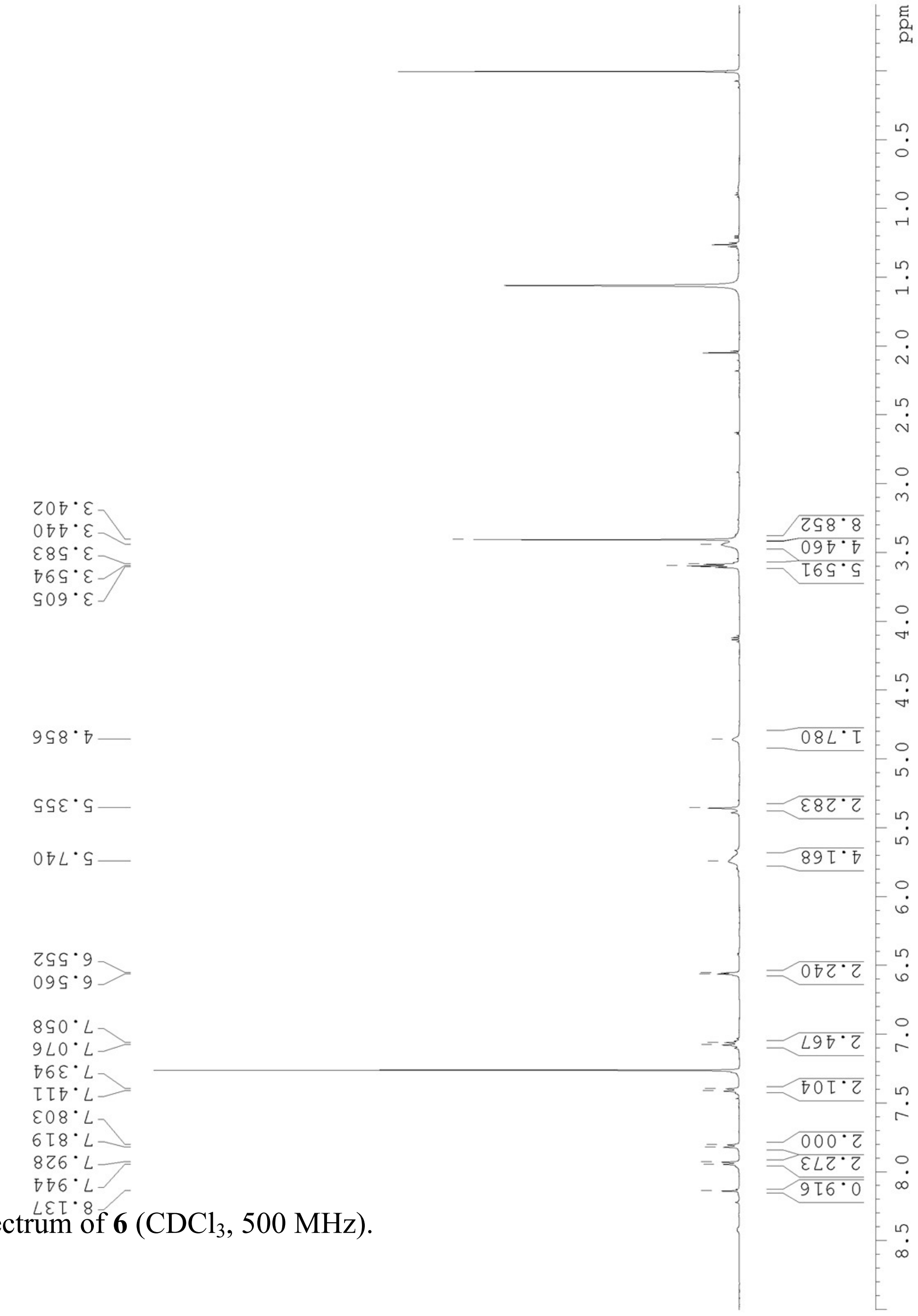

**Figure S8.** $^1$H NMR spectrum of **6** ($CDCl_3$, 500 MHz).

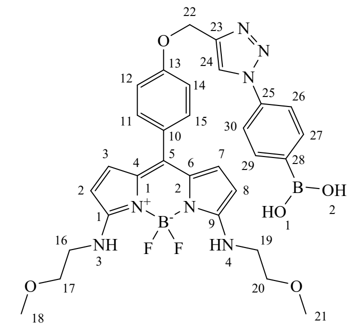

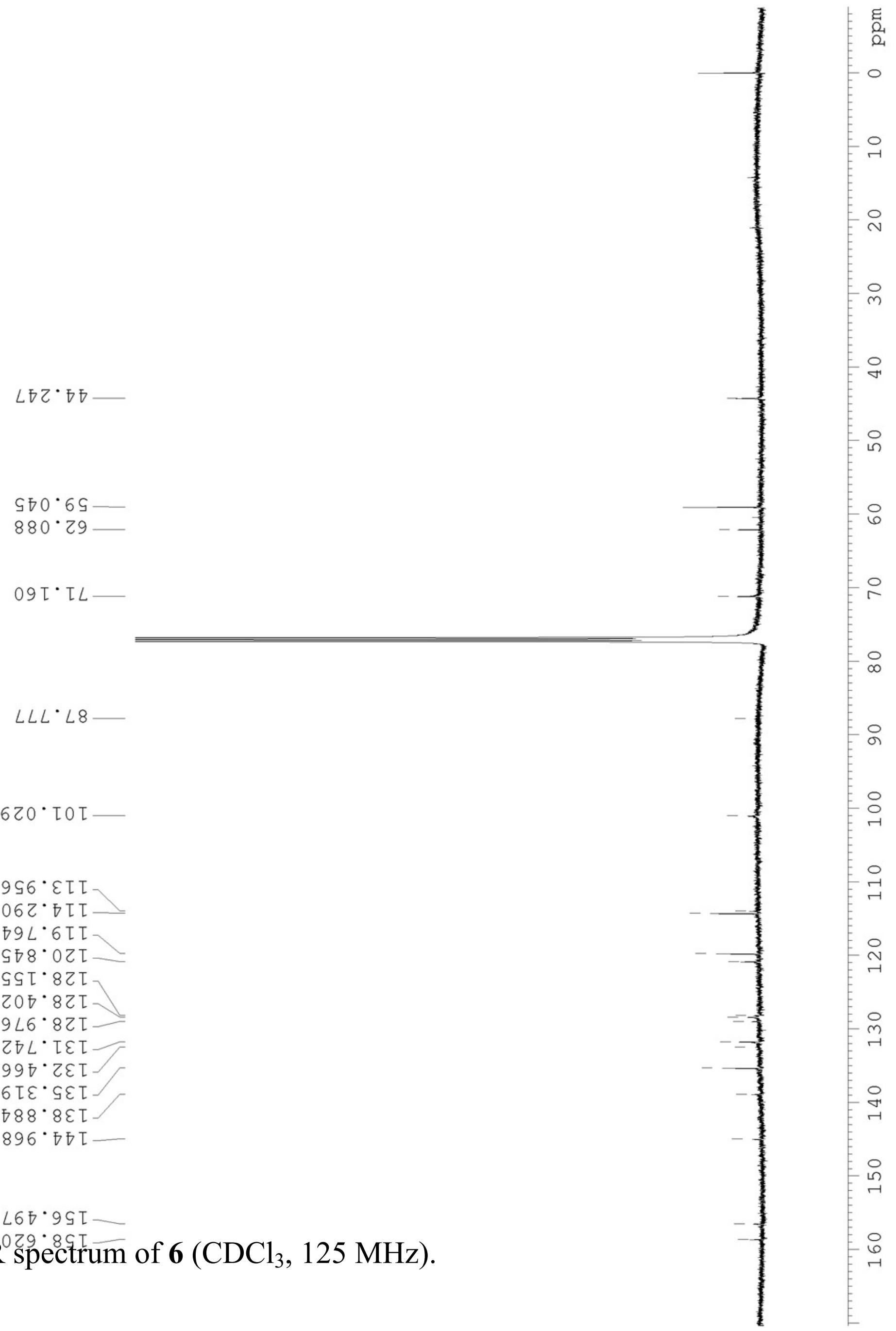


**Figure S9.** $^{13}C\{^{1}H\}$ NMR spectrum of **6** ($CDCl_3$, 125 MHz).

Table S1: Effect of interference substances on fructose assay

| sample | $\Delta F \pm$ SE ($n$ = 3) |
|---|---|
| 2 mM D-fructose | 361 ± 29 |
| + 1 mM uric acid | 389 ± 10 |
| + 1 mM ascorbic acid* | 424 ± 15 |
| + 0.5%(v/v) ethanol | 375 ± 7 |
| + 0.5 mg/mL hen egg lysozyme | 383 ± 10 |
| + 0.5 mg/mL chicken ovalbumin* | 892 ± 13 |

*ANOVA followed by Dunnett's test showed that the changes after the addition of interference substances are statistically significant ($P < 0.05$).
